# Gallbladder and small bowel metastasis of regressive melanoma: a case report

**DOI:** 10.1259/bjrcr.20180032

**Published:** 2018-08-01

**Authors:** Giulia R Ercolino, Giuseppe Guglielmi, Luca Pazienza, Filomena Urbano, Diego Palladino, Anna Simeone

**Affiliations:** 1 Department of Radiology, University of Foggia, Foggia, Italy; 2 Department of Radiology, Scientific Institute Hospital “Casa Sollievo della Sofferenza”, San Giovanni Rotondo, Italy

## Abstract

Malignant melanoma development in gastrointestinal (GI) tract may be primary or secondary. Although small bowel, colon and stomach represent common GI sites affected from metastatic cutaneous malignant melanoma (cMM), more than 90% of the cases are identified only during autoptic examinations. Therefore, the diagnosis in a living patient of gallbladder metastasis from cMM is considered extremely rare. We aimed to describe a case of metastatic melanoma involving the gallbladder, the stomach and the small bowel in a 78-year-old male with diffuse abdominal pain and a history of cMM of the back, which was radically resected 4 years before. Abdominal ultrasound showed intracholecystic multiple nodulations. CT, besides confirming the gallbladder nodules, revealed multiple masses in the stomach, duodenum and ileum. Malignant melanoma lesions were confirmed by histopathological and immunohistochemical analyses of bioptic material obtained from endoscopic examination. In patients with history of melanoma, careful inspection of GI tract should be prompted adopting adequate imaging techniques and endoscopy in order to better influence treatment planning and prognosis.

## Introduction

Malignant melanoma of gastrointestinal (GI) tract, either primary or metastatic, is an uncommon entity that usually remains undiagnosed in living patients, probably as a result of an asymptomatic course or nonspecific symptoms.^1, 2^ Small bowel, colon and stomach are the commonest sites of GI metastasis from cutaneous malignant melanoma (cMM) and basically represent an autoptic finding. The gallbladder involvement is rare, usually inscribed in a context of diffuse metastatic disease, and its description in a living patient has been barely described in literature.^3, 4^ We describe a case of 78-year-old male with metastasis of gallbladder, stomach and small bowel from cutaneous primary malignant melanoma.

## Case presentation

**Figure 1.**
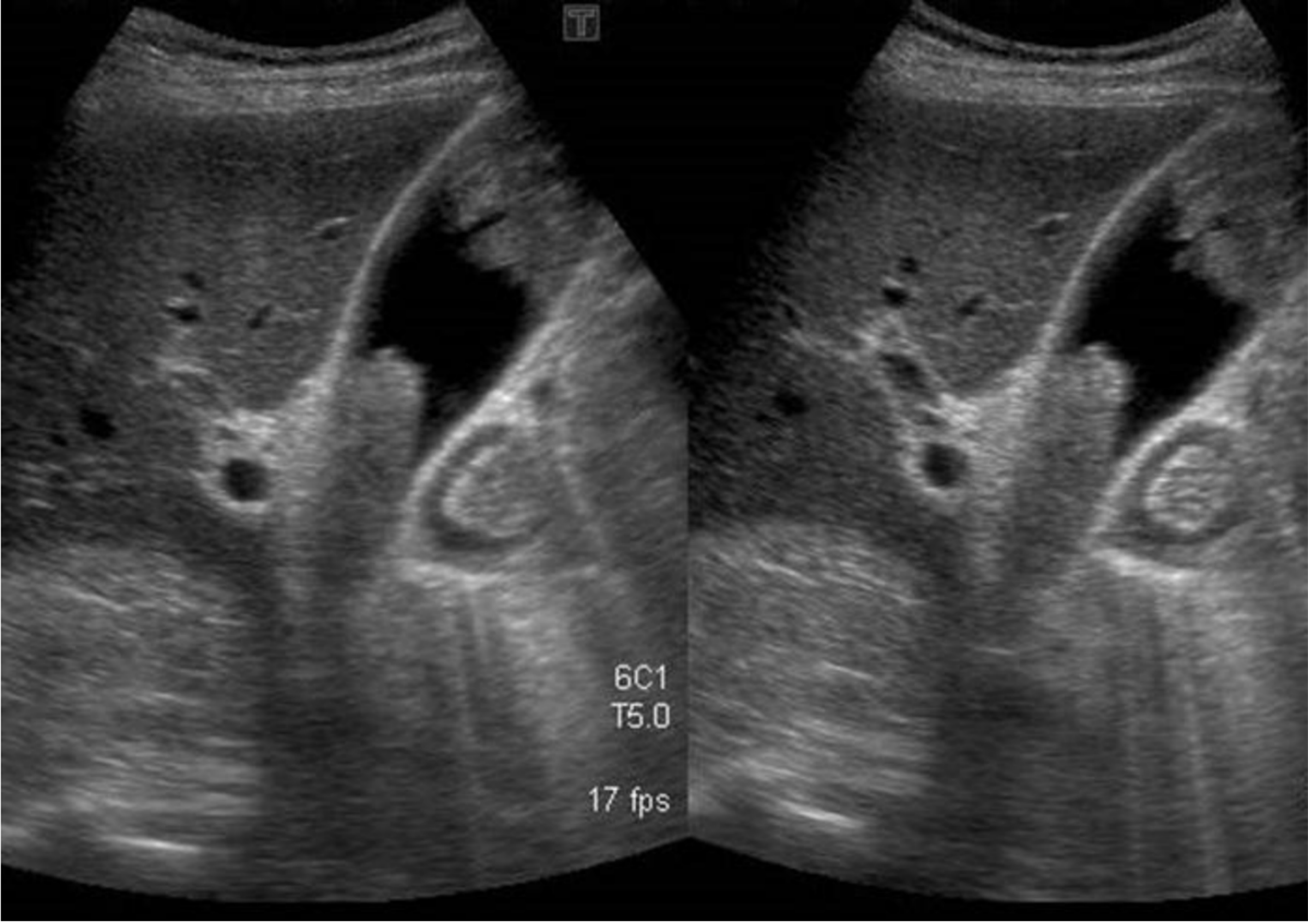
Hepatobiliary ultrasound. Hyperechoic masses involving the gallbladder fundus, body and neck; the parietal lesions with minimal to absent acoustic shadowing.

**Figure 2.**
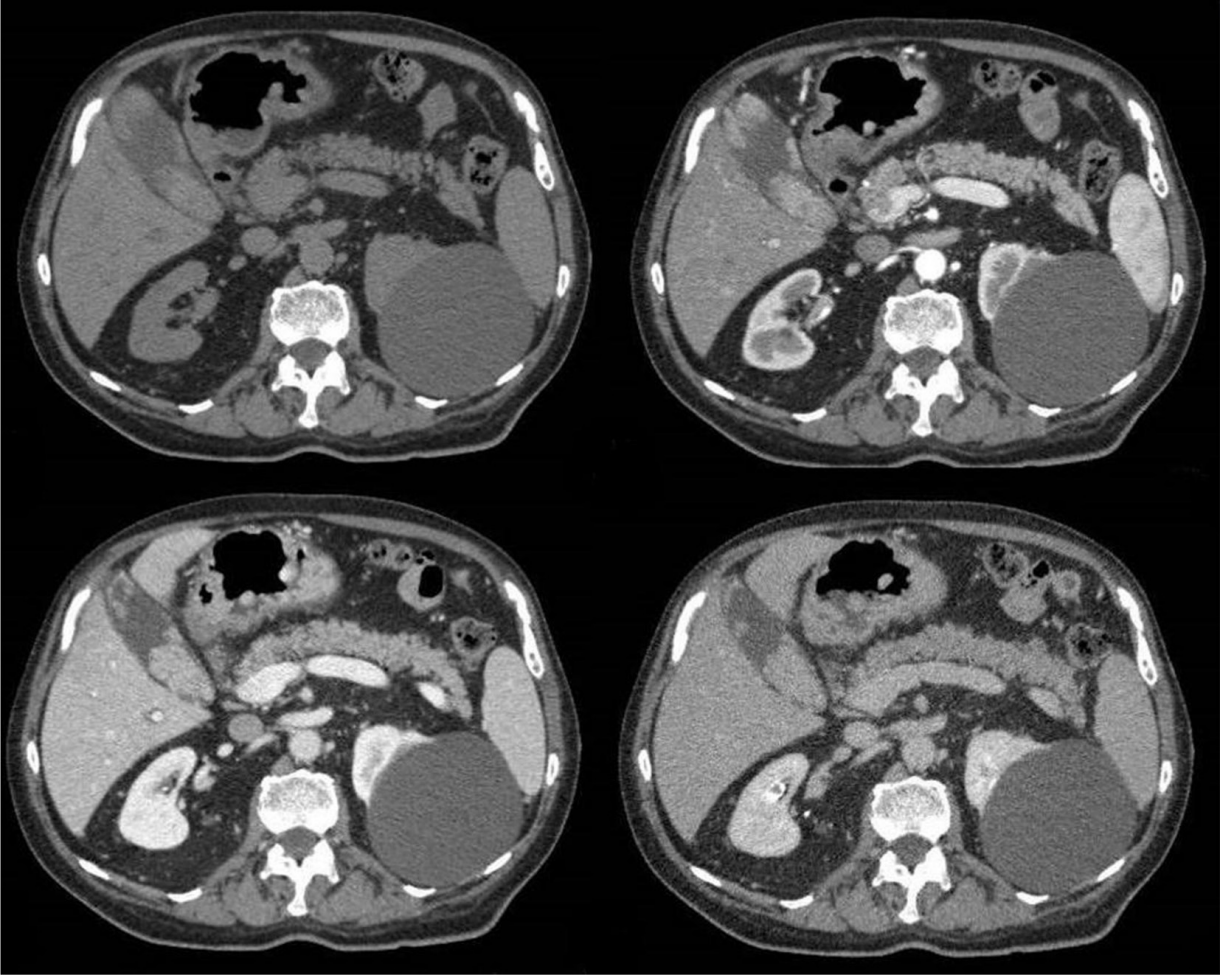
(a–d) CECT, contrast-enhanced CT of the abdomen . Multiple nodular lesions in the gallbladder show early intense enhancement followed by progressive washout .

**Figure 3.**
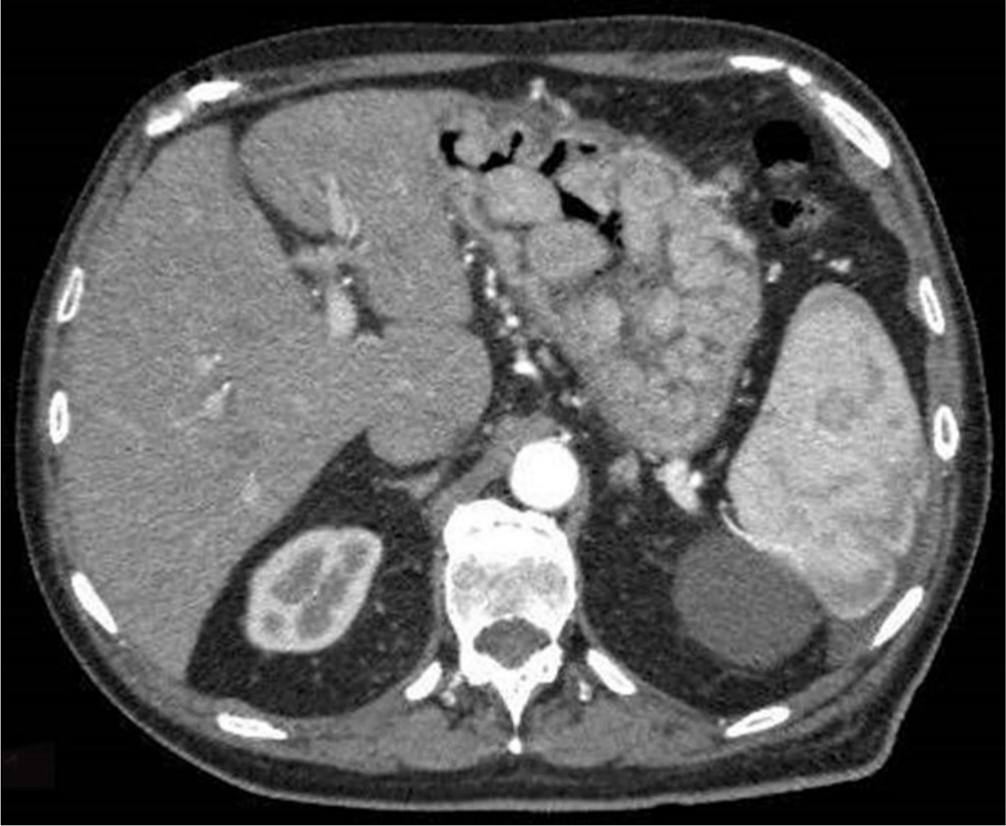
Abdominal CECT. Multiple solid masses in the stomach with early intense enhancement followed by progressive washout. CECT, contrast enhanced CT

**Figure 4.**
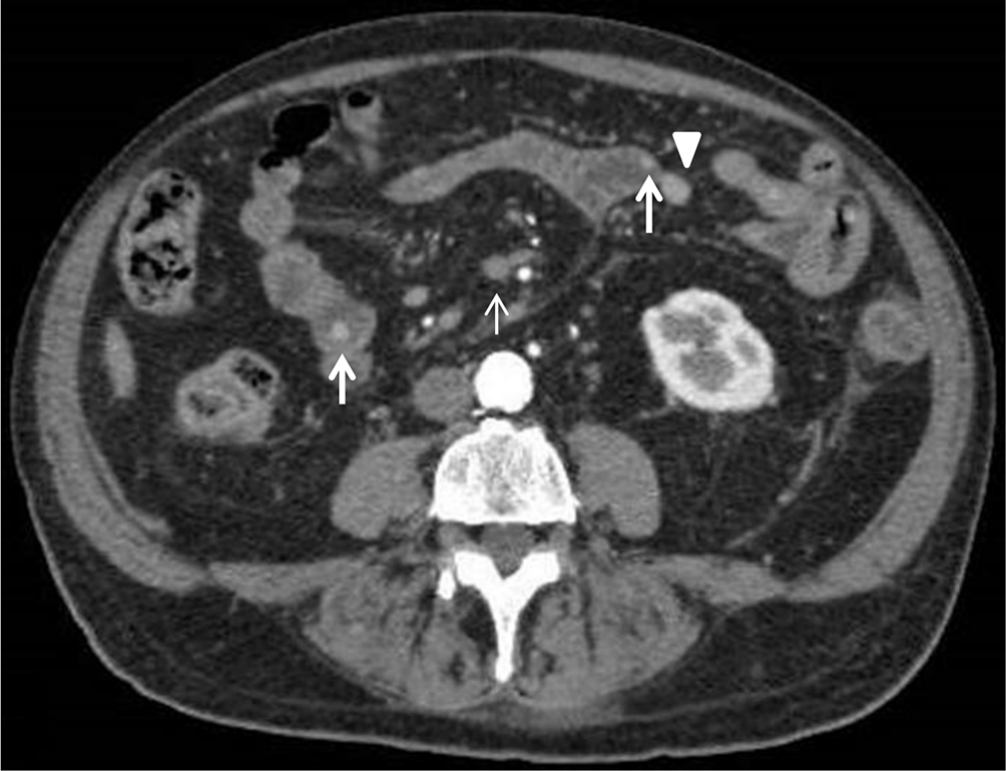
Abdominal CECT. Nodules in small bowel (arrows) with early intense enhancement followed by progressive washout. Peritoneal nodules (arrowhead) and mesenteric lymph nodes (thin arrow). CECT, contrast enhanced CT.

**Figure 5.**
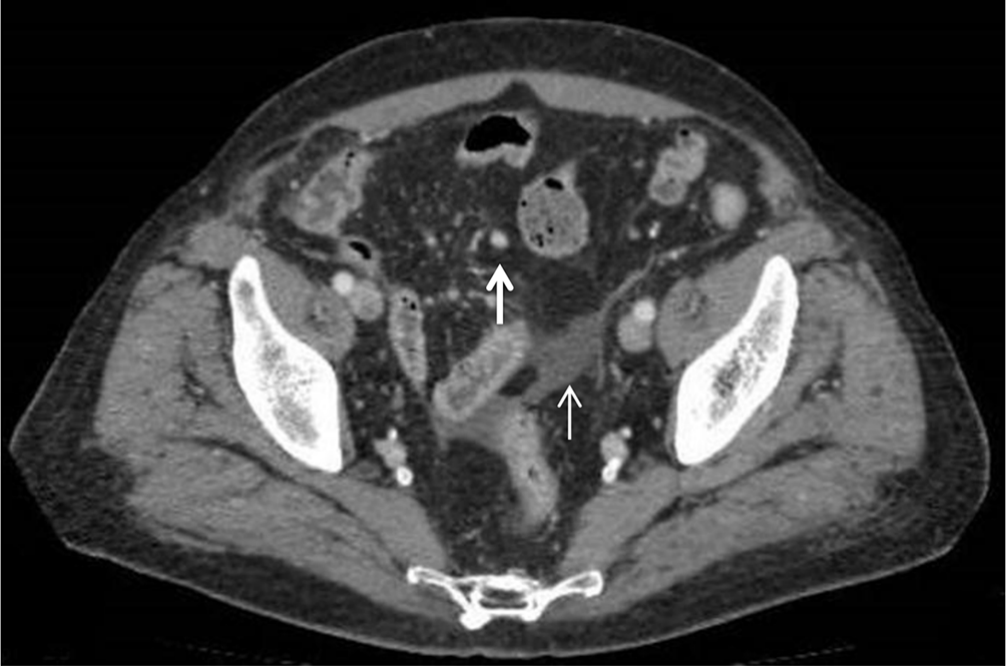
Abdominal CECT. Mesenteric lymph nodes (arrow) and pelvis ascites (thin arrow). CECT, contrast enhanced CT.

**Figure 6.**
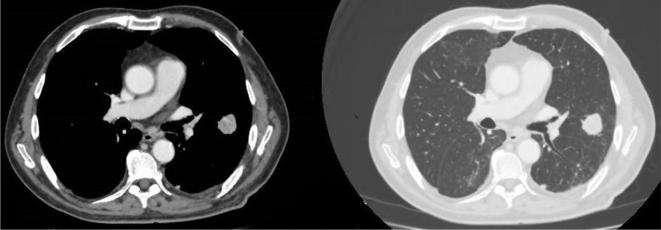
Thoracic CT. Pulmonary, solid, and bilateral nodules with no pleural effusion.

A 78-year-old male came to Scientific Institute Hospital “Casa Sollievo della Sofferenza” for diffuse abdominal pain. His past medical history revealed a total excision of a dorsal cMM 4 years before with no evidence of metastatic disease at the time of diagnosis. Physical examination of the patient appeared to be good in terms of general health and nutritional status: abdomen was soft, with no palpable masses. There was no evidence of melaena and laboratory results were normal. The hepatobiliary ultrasound evidenced multiple intracholecystic nodules involving the gallbladder fundus, body and neck; the parietal lesions appeared like hyperechoic masses of variable sizes (≥1 cm), with minimal to absent acoustic shadowing. The rest of abdomen was poorly explorable due to abdominal adiposity (Figure 1). Contrast-enhanced CT (CECT) of the abdomen confirmed the gallbladder parietal lesions and revealed multiple solid masses (up to 5 cm) with early contrast-enhancement (CE) and progressive washout located in the stomach, duodenum and ileum. Diffuse peritoneal nodules, mesenteric lymph nodes and pelvic ascites were detected, too (Figures 2–5). Chest CT scan showed bilateral solid pulmonary nodules with no pleural effusion (Figure 6). The possibility of metastatic lesions was considered. Esophagogastroduodenoscopy described multiple, friable, parietal masses of stomach and duodenum with melanosis. Histopathological and immunohistochemical analyses confirmed metastatic melanoma in bioptic material and the patient underwent palliative treatment.

## Discussion

Melanoma is a malignant tumor arising from pigment-containing cells, known as melanocytes, which are mainly located in the cutaneous tissue. Primary tumors commonly occur on the skin (over 90%), and have a strong association with excessive sunlight exposure. However. they can also develop from other tissues containing melanocytes such as meninges, GI, mucosa and eyes.^4, 5^ The incidence of cMM is rapidly increasing in Europe and USA; according to recent reports, approximately 13,2000 new cases of melanoma are globally diagnosed each year and the mortality still remains high.^4^ According to post-mortem records, GI metastasis from cMM are not uncommon (50–60% of cadaveric studies in MM patients) and usually represent the expression of an advanced and widespread disease; small bowel, colon and stomach are the most frequent localizations, while the gallbladder has been described in 4–20% of cadavers.^3, 4^ Considering that only 1–9% of overall GI metastasis from cMM are diagnosed ante-mortem, the identification of gallbladder metastasis from cMM in a living patient is extremely rare.^4, 6,7^

Melanoma is a malignant tumor arising from pigment-containing cells, known as melanocytes, which are mainly located in the cutaneous tissue. Primary tumors commonly occur on the skin (over 90%), and have a strong association with excessive sunlight exposure. However. they can also develop from other tissues containing melanocytes such as meninges, GI, mucosa and eyes.^4, 5^ The incidence of cMM is rapidly increasing in Europe and USA; according to recent reports, approximately 13,2000 new cases of melanoma are globally diagnosed each year and the mortality still remains high.^4^ According to post-mortem records, GI metastasis from cMM are not uncommon (50–60% of cadaveric studies in MM patients) and usually represent the expression of an advanced and widespread disease; small bowel, colon and stomach are the most frequent localizations, while the gallbladder has been described in 4–20% of cadavers.^3, 4^ Considering that only 1–9% of overall GI metastasis from cMM are diagnosed ante-mortem, the identification of gallbladder metastasis from cMM in a living patient is extremely rare.^4, 6,7^

GI metastasis normally have an asymptomatic course or non-specific symptoms; clinical presentation is related to complications such as hemorrhage, obstruction, perforation, intussusception, vomiting, weight loss and, rarely, cholecystitis in case of gallbladder involvement.^6, 7^ Imaging is crucial for the diagnosis of metastatic disease: secondary lesions can appear as flat and infiltrative or as polypoid lesions.^3, 8^ Ultrasound is the first technique for abdomen investigation and is capable of detecting intra cholecystic nodules, gastrointestinal masses and parietal thickening. Ultrasound aspects include single or multiple hyperechoic masses with minimal to absent acoustic shadowing probably due to their low density.^9^ Biliary ducts can present dilation, most often the common hepatic duct. On basic CT scan lesions can appear as isodense to hyperdense (compared to muscle density) nodules protruding into gastrointestinal tract; due to their hypervascularity, on CECT they often show intense enhancement in early arterial phase followed by progressive wash-out.^3, 10^ On MRI, lesions typically show T1 signal hyperintensity and T2 signal hypointensity. Since lesions may be obscured by the enhancing biliary and gallbladder mucosa, gadolinium contrast administration is not necessarily helpful. Magnetic resonance cholangiopancreatography and endoscopic retrograde cholangiopancreatography aspects include polypoid filling defects or irregular narrowing of the extrahepatic duct.^11^ GI endoscopy can identify three different types of melanoma lesions: the ulcerated mucosal masses, the necrotic lesions with melanosis and the amelanotic lesions.^4^ The differential diagnosis for gallbladder melanoma include adenocarcinoma, metastatic disease and cholesterol polyps. Malignant polypoid lesions usually measure larger than 1 cm in diameter and compared with benign lesions they show early and progressive washout. Unlike tumefactive sludge or stone, melanoma metastasis are vascularized and without mobility.^11^ Management options depend on the extension of disease in terms of location and number of lesions. The prognosis of metastatic melanoma of the gallbladder is very poor, with survival rate of 8.4 months.^3, 4^

## Conclusions

Although GI involvement in metastatic melanoma is not uncommon, it usually has an asymptomatic course and more than 90% of the cases remain undiagnosed ante-mortem. Indeed, the diagnosis of gallbladder involvement in a living patient is extremely rare. In patients with history of melanoma, careful inspection of GI tract should be prompted adopting adequate imaging techniques and endoscopy in order to better influence treatment planning and prognosis.

## Learning points

- Gastrointestinal (GI) malignant melanoma may be primary or secondary.
- The most common sites of GI metastases from cutaneous malignant melanoma are small bowel, colon and stomach.
- GI malignant melanoma shows an asymptomatic course and more than 90% of the cases remain undiagnosed ante-mortem.
- The diagnosis of gallbladder involvement in a living patient is extremely rare.
- In patients with history of melanoma, careful inspection of GI tract should be prompted adopting adequate imaging techniques and endoscopy in order to better influence treatment planning and prognosis.
